# Disease Control and Manageable Toxicity With Epcoritamab in Refractory Diffuse Large B‐Cell Lymphoma‐Type Richter Syndrome: A Case Report in an Elderly Patient

**DOI:** 10.1002/cnr2.70349

**Published:** 2025-09-16

**Authors:** Takumi Nishikawa, Masuho Saburi, Shogo Urabe, Eiichi Ohtsuka

**Affiliations:** ^1^ Department of Hematology Oita Prefectural Hospital Oita Japan; ^2^ Department of Pathology Oita Prefectural Hospital Oita Japan

**Keywords:** chronic lymphocytic leukemia, diffuse large B‐cell lymphoma, epcoritamab, Richter syndrome, small lymphocytic lymphoma

## Abstract

**Background:**

Richter syndrome (RS) is an aggressive transformation of chronic lymphocytic leukemia (CLL)/small lymphocytic lymphoma (SLL) with poor prognosis. No standard treatment for RS has been established. Epcoritamab, a CD3xCD20 bispecific antibody, may offer clinical benefit.

**Case:**

An 80‐year‐old woman was diagnosed with SLL in 2017 and remained untreated for nearly 6 years. In 2023, she developed a bulky abdominal tumor. Although the initial biopsy showed SLL progression without transformation, subsequent treatments with acalabrutinib, venetoclax plus rituximab, and bendamustine plus rituximab failed. In 2024, transformation to diffuse large B‐cell lymphoma‐type RS (DLBCL‐RS) was confirmed. Two lines of cytotoxic regimens were ineffective, with rapid tumor growth. Epcoritamab was initiated as third‐line therapy. Grade 1–3 cytokine release syndrome occurred but resolved with standard management. No neurotoxicity was observed. Partial response (PR) was achieved by week 12, and the patient has maintained PR for 10 months. Infections including COVID‐19 and cytomegalovirus were managed successfully, and immunoglobulin replacement was introduced for hypogammaglobulinemia.

**Conclusion:**

This case highlights the potential of epcoritamab as an important treatment option for elderly patients with refractory DLBCL‐RS.

## 1 Introduction

Richter syndrome (RS) is defined as a histologically confirmed diagnosis of diffuse large B‐cell lymphoma (DLBCL) or Hodgkin's lymphoma (HL) in patients with chronic lymphocytic leukemia (CLL)/small lymphocytic lymphoma (SLL). No standard treatment for RS has been established. Thus, most patients with RS are treated with combination chemotherapies used for de novo DLBCL or HL. The outcomes of conventional chemotherapies for patients with DLBCL‐type RS (DLBCL‐RS) are especially poor, with complete response (CR) rates of 20% and median overall survival (OS) ranging from 6 to 12 months [1, 2]. Epcoritamab is a CD3xCD20 T‐cell engaging, bispecific antibody that activates and directs T‐cells to kill malignant CD20‐positive B‐cells. Epcoritamab is expected to have efficacy against DLBCL‐RS, but there are few reports about this due to the rarity of RS. We report an elderly patient with refractory DLBCL‐RS, treated with epcoritamab, resulting in a marked reduction of a bulky tumor with manageable toxicity.

## 2 Case

**FIGURE 1:**
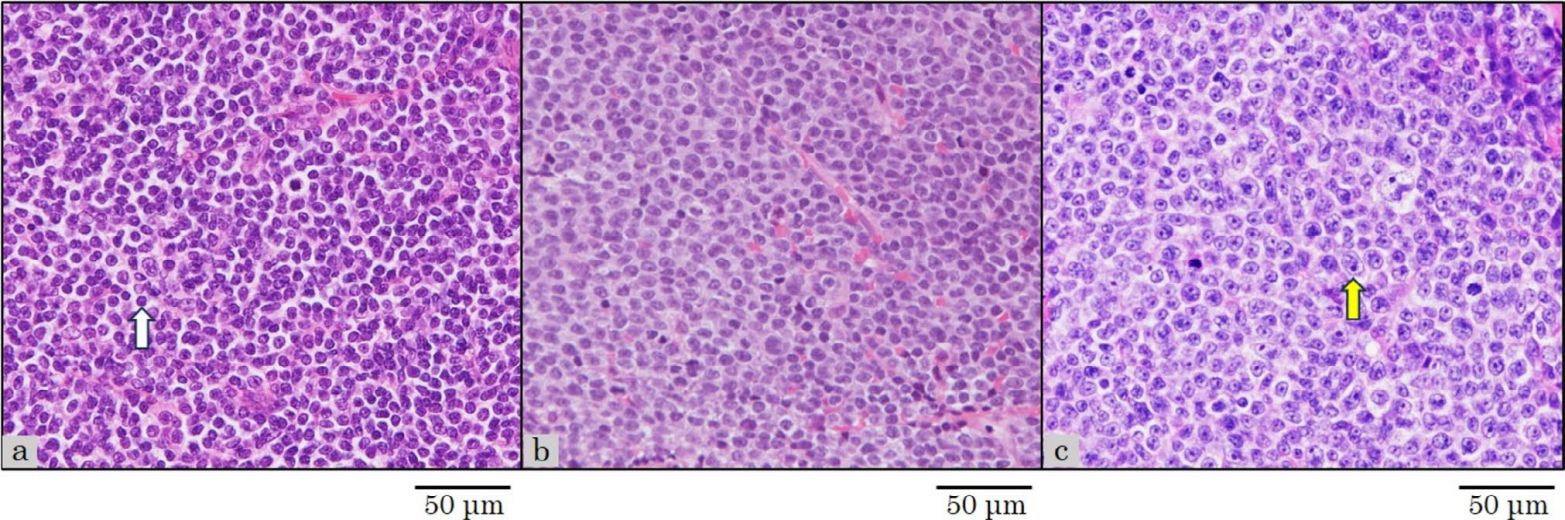
Histopathological findings of a left axillary lymph node at the time of diagnosis of small lymphocytic lymphoma (SLL) (a), bulky abdominal tumor before acalabrutinib as first‐line chemotherapy (b), and left axillary lymph node at the time of diagnosis of diffuse large B‐cell lymphoma (DLBCL) (c) on hematoxylin and eosin staining (×400). Diffuse proliferation of small to medium‐sized, atypical lymphoid cells (white arrow) is observed in the left axillary lymph node at the time of diagnosis of SLL (a) and a bulky abdominal tumor before primary chemotherapy (b). Diffuse proliferation of medium to large‐sized, atypical lymphoid cells (yellow arrow) is observed in a left axillary lymph node at the time of diagnosis of DLBCL (c).

**FIGURE 2:**
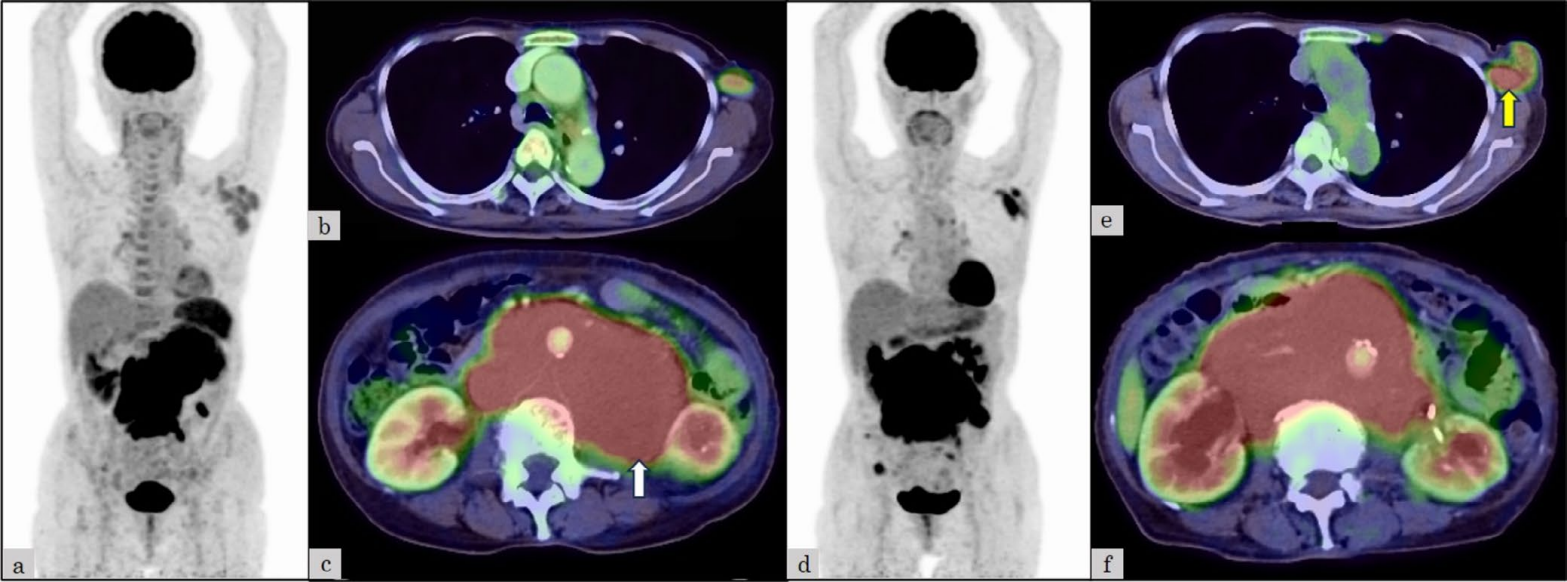
Computed tomography (CT) and ^18^F‐fluorodeoxyglucose (FDG) positron emission tomography/CT (FDG‐PET/CT) images. In May 2023, FDG‐PET/CT showed a bulky abdominal tumor and splenomegaly, both with high FDG uptake (SUVmax: 10.0 in the abdominal tumor and 5.1 in the spleen), as well as mild FDG uptake in the left axillary lymphadenopathy (SUVmax: 3.3) (a–c). A CT‐guided needle biopsy was performed at the location indicated by the white arrow in (c), and a histopathological diagnosis of small lymphocytic lymphoma was established. In June 2024, FDG‐PET/CT showed a bulky abdominal tumor and left axillary lymphadenopathy, both with high FDG uptake (SUVmax: 15.6 in the abdominal tumor and 7.6 in the axillary node) (d–f). A biopsy of the left axillary lymph node was performed at the site indicated by the yellow arrow in Fig. e, and the histopathological diagnosis was diffuse large B‐cell lymphoma.

**FIGURE 3:**
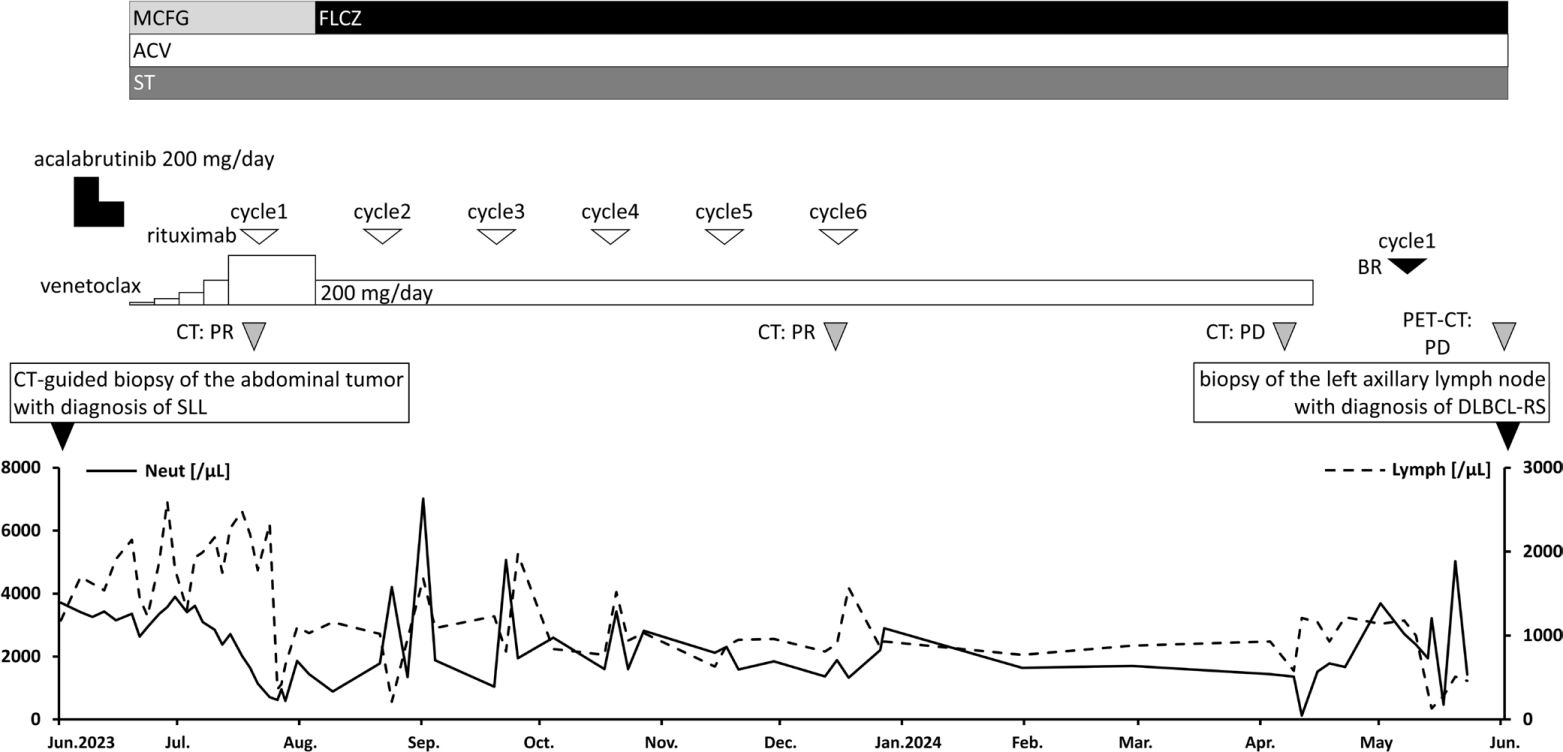
Clinical course and treatment of small lymphocytic lymphoma. Acalabrutinib was started in June 2023 but was discontinued 2 weeks later due to intolerance and PD. VenR led to a reduction in the abdominal tumor; however, it resulted in PD 10 months after the start of VenR. Subsequent third‐line therapy with BR also resulted in PD. ACV, acyclovir; BR, bendamustine and rituximab; CT, computed tomography; DLBCL, diffuse large B‐cell lymphoma; FLCZ, fluconazole; MCFG, micafungin; PD, progressive disease; PET, positron emission tomography; PR, partial response; RS, Richter syndrome; SLL, small lymphocytic lymphoma; ST, sulfamethoxazole trimethoprim; VenR, venetoclax and rituximab.

**FIGURE 4:**
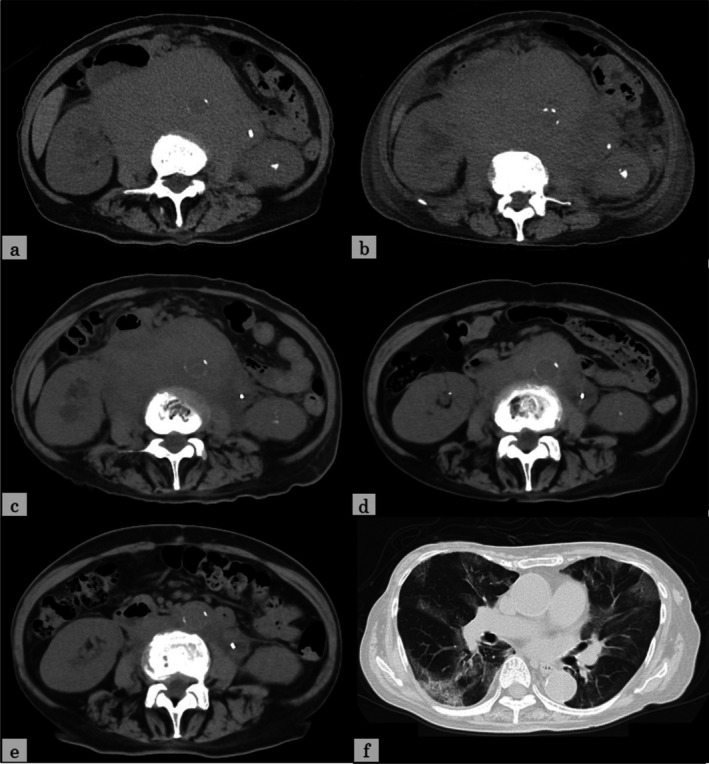
Computed tomography (CT) images. The bulky abdominal tumor shows a slight temporary increase after starting epcoritamab, but then it shows a marked decrease (a: before epcoritamab, b: 4 weeks after starting epcoritamab, c: 8 weeks after starting epcoritamab, d: 12 weeks after starting epcoritamab, e: 5 months after starting epcoritamab). COVID‐19 pneumonia develops just before the final dose of the fourth cycle of epcoritamab (f).

**FIGURE 5:**
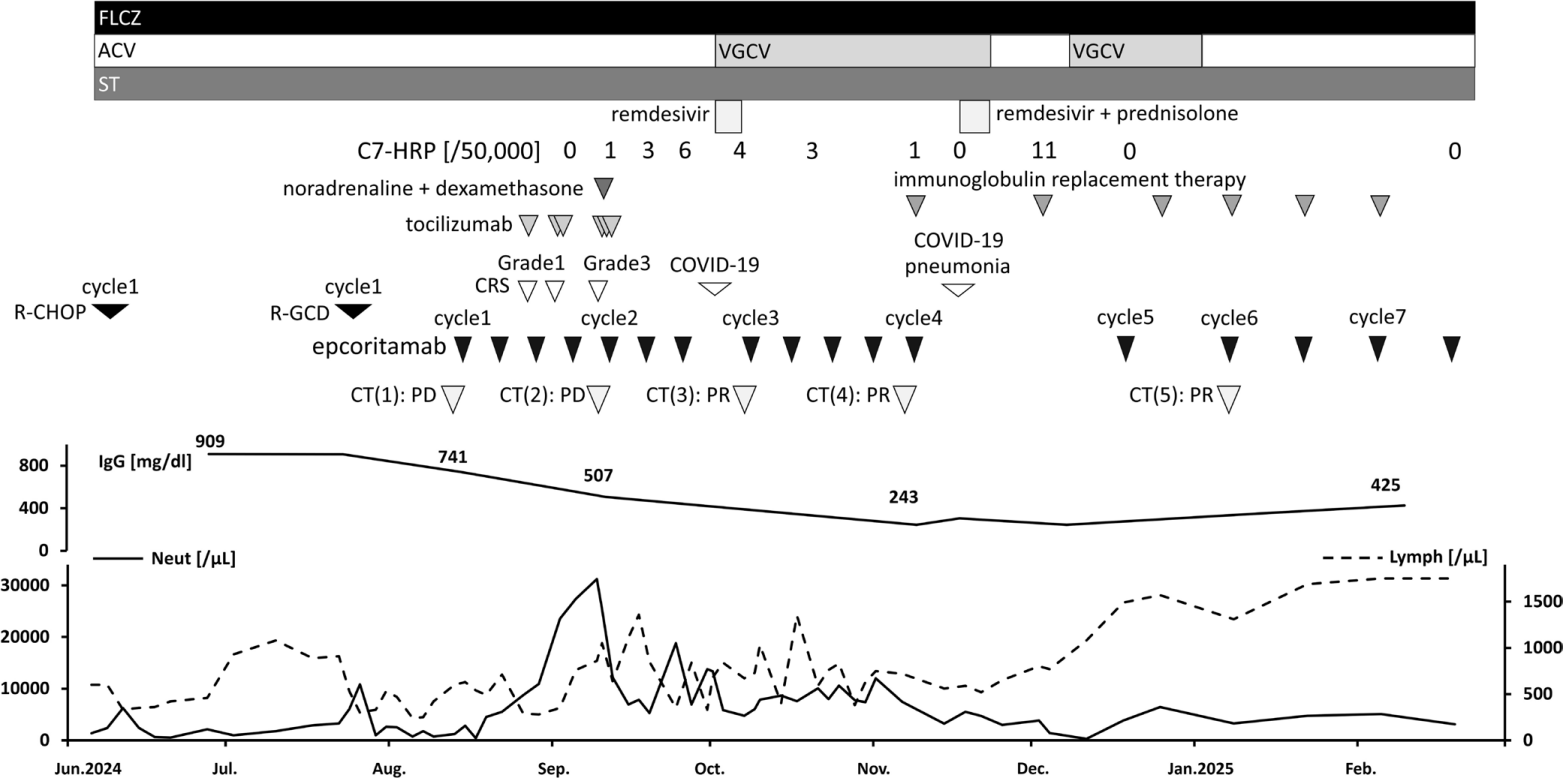
Clinical course and treatment of Richter syndrome. DLBCL‐RS progressed after one cycle each of R‐CHOP and R‐GCD. In August 2024, epcoritamab was initiated as third‐line therapy. CT scans were performed at the following time points relative to epcoritamab administration: (1) before epcoritamab, (2) 4 weeks, (3) 8 weeks, (4) 12 weeks, and (5) 5 months after epcoritamab. The corresponding response assessments are also shown in the figure. Grade 1–3 CRS occurred but was successfully managed with tocilizumab, dexamethasone, and noradrenaline. No neurotoxicity was observed. Despite slight tumor enlargement and elevated LDH at week 4, treatment was continued based on an indeterminate response. By week 12, tumor volume had decreased by ≥ 50% and achieved PR. During treatment, the patient developed COVID‐19 infection, CMV antigenemia, and hypogammaglobulinemia, all of which were appropriately managed with antivirals, corticosteroids, and immunoglobulin replacement therapy. ACV, acyclovir; C7‐HRP, cytomegalovirus pp65 antigen; CMV, cytomegalovirus; CRS, cytokine release syndrome; CT, computed tomography; DLBCL, diffuse large B‐cell lymphoma; FLCZ, fluconazole; PD, progressive disease; PR, partial response; R‐CHOP, rituximab, cyclophosphamide, doxorubicin, vincristine, and prednisolone; R‐GCD, rituximab, gemcitabine, carboplatin, and dexamethasone; RS, Richter syndrome; ST, sulfamethoxazole trimethoprim; VGCV, valganciclovir.

An 80‐year‐old woman presented with left axillary lymphadenopathy and visited Oita Prefectural Hospital in August 2017. Computed tomography (CT) showed only left axillary lymphadenopathy with a maximum diameter of 25 mm, and no abnormalities were detected on blood tests. Pathological examination of a biopsied left axillary lymph node showed diffuse proliferation of small to medium‐sized atypical lymphoid cells (Figure 1a), positive for CD20, CD79a, CD5, CD23, and bcl‐2 and negative for CD10, cyclin D1, and CD3. Chromosome analysis was normal karyotype, and del17p was unevaluated. SLL was diagnosed, but treatment was not indicated according to the International Workshop on Chronic Lymphocytic Leukemia criteria [3]. The SLL was observed without treatment for nearly 6 years. In May 2023, ^18^F‐fluorodeoxyglucose (FDG) positron emission tomography/CT (FDG‐PET/CT) revealed a bulky abdominal tumor, measuring 122 × 86 mm^2^ and splenomegaly, both with high FDG uptake (SUVmax: 10.0 in the abdominal tumor and 5.1 in the spleen). Additionally, cervical, supraclavicular, and left axillary lymphadenopathies showed mild FDG uptake (SUVmax: 3.3) (Figure 2a–c). The abdominal tumor was compressing the left ureter, necessitating the placement of a ureteral stent. CT‐guided needle biopsy of the bulky abdominal tumor showed proliferating small lymphocytes with mild atypia (Figure 1b), positive for CD20, CD79a, CD5, and CD23 and negative for CD3. The Ki‐67 index of tumor cells was 20%. Pathological examination showed progression of SLL, not histological transformation. Fluorescence in situ hybridization (FISH) was positive for del17p. In June 2023, acalabrutinib, a Bruton tyrosine kinase inhibitor, was initiated (Figure 3). However, enlargement of the abdominal tumor was observed within 2 weeks, and the treatment was discontinued due to progressive disease (PD). The patient was subsequently switched to a combination of venetoclax and rituximab (VenR) as second‐line therapy. Five months after the initiation of VenR, the abdominal tumor had decreased in size to 50 × 25 mm^2^, but the disease progressed 10 months after treatment initiation. Third‐line therapy with bendamustine and rituximab (BR) for CLL was then administered, but the disease progressed after the first cycle. In June 2024, FDG‐PET/CT showed a bulky abdominal tumor and the left axillary lymphadenopathy, both with high FDG uptake (SUVmax: 15.6 in the abdominal tumor and 7.6 in the axillary node) (Figure 2d–f). Serum LDH levels were within the normal range. Pathological examination of a left axillary lymph node showed diffuse proliferation of medium to large‐sized atypical lymphoid cells (Figure 1c), positive for CD20, CD79a, CD5, MUM‐1, c‐MYC, and bcl‐2 and negative for CD10, SOX11, and LEF1. The Ki‐67 index of tumor cells was 60%. Chromosome analysis showed complex karyotype (43, X, −X, add(1)(p11), −3, −7, −8, −9, −10, −14, −16, −17, +6mar), and del17p was unevaluated. FISH was negative for the fusion signal of MYC/IgH and BCL2/IgH and the split signal of BCL6. Pathological examination showed DLBCL (non‐germinal center B‐cell type), and a diagnosis of DLBCL‐RS was made. Despite the administration of one cycle each of rituximab, cyclophosphamide, doxorubicin, vincristine, and prednisolone (R‐CHOP) and rituximab, gemcitabine, and carboplatin (R‐GCD) as first‐ and second‐line therapies for DLBCL‐RS, both the bulky abdominal tumor and the left axillary lymph nodes progressed, with the abdominal tumor increasing in size to 143 × 112 mm^2^ (Figure 4a). Due to the rapid tumor progression during cytotoxic chemotherapy, epcoritamab was initiated as third‐line treatment in August 2024 (Figure 5). Grade 1 cytokine release syndrome (CRS) developed after 0.8 mg of epcoritamab and the first administration of 48 mg of epcoritamab, and grade 3 CRS developed after the second administration of 48 mg of epcoritamab. CRS resolved with tocilizumab, dexamethasone, and noradrenaline. Immune effector cell‐associated neurotoxicity syndrome (ICANS) was not observed. Four weeks after starting epcoritamab, the bulky abdominal tumor showed slight enlargement with a partial decrease in the CT attenuation value within the tumor (Figure 4b), whereas serum LDH levels increased to 426 U/L (normal range, 124–222 U/L). The patient continued epcoritamab because the response was classified as an indeterminate response based on the Lymphoma Response to Immunomodulatory Therapy Criteria [4]. Eight weeks after starting epcoritamab, abdominal tumor volume decreased slightly (Figure 4c). Fever recurred just before the final dose of the second cycle of epcoritamab, accompanied by a positive COVID‐19 polymerase chain reaction (PCR) and cytomegalovirus (CMV) antigenemia. With a two‐week interruption of epcoritamab, treatment with remdesivir and valganciclovir resulted in fever resolution and negative CMV antigenemia. Twelve weeks after starting epcoritamab, a tumor volume reduction of at least 50% was confirmed (Figure 4d), and a partial response was maintained at 5 months (Figure 4e). From the fourth cycle, immunoglobulin replacement therapy was started for hypogammaglobulinemia. Just before the final dose of the fourth cycle of epcoritamab, the patient developed COVID‐19 pneumonia (Figure 4f), which improved with remdesivir, prednisolone, and a six‐week treatment interruption of epcoritamab. Immunoglobulin replacement therapy maintained serum IgG levels above 400 mg/dL without subsequent infections. Epcoritamab has been continued for 10 months with no other notable adverse or unexpected events. The patient remains alive with a partial response (PR) at the time of the last observation.

## 3 Discussion

RS is a rare but aggressive histological transformation of CLL/SLL, and the most common transformation is to DLBCL [5]. Most patients with DLBCL‐RS are treated with combination chemotherapy regimens used for de novo DLBCL, but outcomes are poor. In a previous single‐center retrospective study of chemotherapy‐based treatment for RS, the clinical response to first‐line therapy was CR in 36% and PR in 24%, with a median OS of 12 months. In that cohort, patients treated with an R‐CHOP‐like regimen had a median OS of 15.3 months, and those treated with platinum‐ or high‐dose cytarabine‐containing regimens had a median OS of 14.6 months [1]. In contrast, a recent multicenter analysis by Kittai et al. reported a median OS of 25.8 months in a modern cohort of patients with RS. However, patients who had received prior BTK inhibitors or BCL2 inhibitors therapy for CLL had significantly worse outcomes, with a median OS of only 12.3 months. Notably, among patients who underwent subsequent therapies, those receiving allogeneic stem cell transplantation achieved a median OS of 54.5 months [6]. Chimeric antigen receptor T‐cell (CAR‐T) therapy and bispecific antibody are important treatment options for relapsed or refractory DLBCL. Since RS has been excluded from the clinical trials of CAR‐T therapy for DLBCL, CAR‐T therapy is not approved for RS in Japan. A retrospective analysis of 69 patients with DLBCL‐RS treated with CAR‐T therapy in real‐world practice showed an overall response rate of 63%, including a CR rate of 46%. Despite encouraging responses, the median progression‐free survival was only 4.7 months and OS was 8.5 months. Grade ≥ 3 CRS occurred in 15.9%, and grade ≥ 3 ICANS in 36.8%. These findings highlight the potential of CAR‐T therapy in DLBCL‐RS, though durability remains limited and is often accompanied by relatively common ICANS [7]. Currently, a phase Ib/II trial of epcoritamab for patients with RS, who have received no more than two prior lines of therapy for RS, is ongoing (EPCORE CLL‐1 Trial, NCT04623541). It demonstrated high response rates in RS‐LBCL, with most responses seen by week 6, and a manageable safety profile with only low‐grade, resolving CRS events [8, 9]. A phase I dose‐escalation study of the other CD3xCD20 bispecific antibody, mosunetuzumab, included five patients with RS, and it showed a manageable safety profile and durable complete responses [10]. Although the inclusion of RS patients was not specified, the phase I/II EPCORE NHL‐1 trial in 157 patients with relapsed or refractory LBCL demonstrated high response rates and durable remissions, with lower response rates observed in those with high tumor burden [11, 12]. Given these findings, the favorable outcome observed in the present case despite bulky disease is notable. Furthermore, 18.5% of patients in the EPCORE NHL‐1 trial were over 75 years old, and grade ≥ 3 CRS occurred in only 2.5%. In our case, grade 3 CRS was manageable and resolved promptly, supporting the potential feasibility of epcoritamab even in elderly RS patients. Other adverse events were manageable, allowing for the continuation of epcoritamab while preserving the patient's performance status.

There were several limitations to this report. First, the observation period following epcoritamab administration was extremely short. In clinical trials of epcoritamab for DLBCL, late responders have also been observed [12], and in previous clinical trials, achievement of CR has been associated with prolonged CR duration. These findings highlight that longer follow‐up is warranted to confirm the durability of this response. Second, the May 2023 biopsy of the bulky abdominal mass showed no histopathologic evidence of transformation despite high FDG uptake (SUVmax 10.0). When PET/CT demonstrates an SUVmax ≥ 5, RS should be suspected and tissue biopsy, preferably from the most FDG avid site, is recommended [13, 14]. This discordance may reflect intralesional heterogeneity and the inherent sampling limitations of core needle biopsy. Third, IGHV sequencing to assess clonal relatedness was not conducted, which has been highlighted as a prognostic factor in RS [5]. In this case, the diagnoses of both SLL and DLBCL‐RS were made on biopsies taken from the same disease site, which was clinically suspected to have undergone histological transformation from SLL to DLBCL. Lastly, the treatment response to acalabrutinib, BR, R‐CHOP, and R‐GCD was assessed after only one cycle or within one month of each therapy, and the short treatment duration may have precluded an accurate evaluation of efficacy.

In conclusion, this case highlights epcoritamab as a potentially valuable treatment option for patients with DLBCL‐RS, particularly those with limited response to conventional chemotherapy. Its manageable safety profile may also support its use in elderly patients who are not eligible for CAR‐T therapy or hematopoietic stem cell transplantation. Given the limited clinical data currently available, further case reports and prospective studies are warranted to clarify the therapeutic potential of epcoritamab in RS.

## Author Contributions

**T.N.** and **M.S.:** conceptualization. **T.N.** and **M.S.:** writing – original draft. **S.U.** and **E.O.:** writing – review and editing.

## Consent

Written informed consent was obtained from the patient.

## Conflicts of Interest

The authors declare no conflicts of interest.

## Data Availability

Data sharing not applicable to this article as no datasets were generated or analyzed during the current study.
